# A real-world pharmacovigilance study of FDA adverse event reporting system events for bictegravir/emtricitabine/tenofovir alafenamide

**DOI:** 10.3389/fphar.2026.1945154

**Published:** 2026-10-15

**Authors:** Ying Liu, Leidan Zhang, Xin Li, Hongxin Zhao

**Affiliations:** Clinical Center for HIV/AIDS, Beijing Ditan Hospital, Capital Medical University, Beijing, China

**Keywords:** adverse drug signals, bictegravir/emtricitabine/tenofovir alafenamide, FDA adverse event reporting system, HIV, pharmacovigilance

## Abstract

**Introduction:**

Bictegravir/emtricitabine/tenofovir alafenamide (B/F/TAF) is a widely used antiretroviral regimen, but its real-world long-term safety profile in diverse populations remains incompletely characterized.

**Methods:**

We analyzed adverse drug signals from the FAERS database (2018–2025) with B/F/TAF as the primary suspected drug. Disproportionality analyses using reporting odds ratio (ROR), proportional reporting ratio (PRR), Bayesian confidence propagation neural network (BCPNN), and multi-item gamma Poisson shrinker (MGPS) algorithms were performed to detect safety signals.

**Results:**

A total of 6,816 case reports and 13,853 adverse events were included. The highest numbers of reports were related to “Injury, poisoning and procedural complications” (n = 1,792), followed by “General disorders and administration site conditions” (n = 1,726) and “Investigations” (n = 1,499). The System Organ Classes (SOCs) meeting all four disproportionality criteria were “Congenital, familial and genetic disorders” and “Pregnancy, puerperium and perinatal conditions.” The most frequently reported preferred terms were weight increase (408 cases), product dose omission (242 cases), and maternal exposure during pregnancy (202 cases), while the strongest signals by ROR were viral load increased (ROR 160.14), CD4 lymphocytes decreased (ROR 124.60), and immune reconstitution inflammatory syndrome (ROR 88.12). Overall, 37.94% of events occurred within the first month of treatment, and 17.49% occurred after one year.

**Conclusion:**

These pharmacovigilance signals merit clinical awareness, particularly regarding metabolic monitoring, virologic/immunologic parameters (which likely reflect treatment response rather than drug toxicity), and pregnancy exposure reporting. Events occurred both early and late, reinforcing the necessity of longitudinal pharmacovigilance for this widely used antiretroviral regimen. However, these findings are exploratory and should not be interpreted as confirmed causal associations.

## 1 Introduction

The global public health issue posed by HIV infection and transmission remains critical. According to statistics, approximately 1.3 million individuals were newly infected with HIV globally in 2024, and around 630,000 succumbed to AIDS-related illnesses. Antiretroviral therapy (ART) care improves outcomes of people living with HIV (PLWH) and reduces transmission. Bictegravir/emtricitabine/tenofovir alafenamide (B/F/TAF) is an integrase-strand transfer inhibitor (INSTI)- based three-drug coformulation consisting of bictegravir (BIC), emtricitabine (FTC) and tenofovir alafenamide (TAF). Although it is considered an extremely potent antiretroviral with not only a high genetic barrier to resistance but also a reduced renal and bone toxicity (Zhao et al., 2022), the widespread use of B/F/TAF globally necessitates a thorough examination of its long-term safety, particularly regarding potential safety signals in various populations.

Although B/F/TAF has demonstrated good tolerability in clinical trials, the most commonly reported adverse events include diarrhea, nausea, and headache, with incidence rates of approximately 5%–6%. In long-term extension studies and real-world cohorts, weight gain, immune reconstitution inflammatory syndrome (IRIS), and changes in renal laboratory parameters (such as serum creatinine elevation) have been consistently observed (Sax et al., 2023). In addition, post-marketing surveillance and pharmacovigilance analyses have raised potential signals of metabolic disturbances, including diabetes mellitus and dyslipidemia, as well as neuropsychiatric events such as insomnia and depressive disorders (Daar et al., 2025; Hoffmann et al., 2020). Given the expanding global use of B/F/TAF, especially in aging populations, patients with polypharmacy, and those with pre-existing comorbidities, a comprehensive evaluation of its long-term safety profile is urgently needed.

The FDA Adverse Event Reporting System (FAERS) is a crucial database for monitoring global drug safety. While several FAERS-based studies have examined B/F/TAF, few have systematically distinguished pharmacological adverse reactions from medication errors or disease monitoring parameters. This study addresses these gaps using a multi-algorithm analysis of the most recent FAERS data with systematic signal classification. We aim to employ the comprehensive real-world data accumulated post-marketing of B/F/TAF to advance its safety monitoring and deeply investigate potential adverse event signals.

## 2 Materials and methods

### 2.1 Data sources

Adverse event reports were extracted from the FAERS database, a publicly accessible system that compiles spontaneous post-marketing safety data. The database is updated quarterly and contains detailed information on patient demographics, drug exposure, reported events, and clinical outcomes. It encompasses patient demographic and administrative information (DEMO), drug information (DRUG), adverse drug reaction information (REAC), patient outcome information (OUTC), information on report sources (RPSR), drug therapy start and end dates (THER) and indications for use/diagnosis (INDI). The FDA publishes FAERS files every quarter (i.e., four files each year). We screened adverse event reports from the first quarter of 2018 to the first quarter of 2025, including only those reports where B/F/TAF was the primary suspected (PS) drug.

### 2.2 Data processing

The database was searched using both generic and brand names, specifically “bictegravir/emtricitabine/tenofovir alafenamide; bictegravir w/emtricitabine/tenofovir alafenamide; bictegravir sodium/emtricitabine/tenofovir alafenamide fumarate” and “biktarvy.” To address potential duplicates arising from quarterly data updates, we followed the FDA’s official deduplication guidelines. Within each unique CASEID, we selected the record with the most recent FDA_DT. When CASEID and FDA_DT were identical, the entry with the largest PRIMARYID was retained to ensure inclusion of the most complete report. This approach removed 847 duplicate reports from the initial dataset of 7,663 reports, yielding 6,816 unique reports for analysis. Missing data were not imputed; cases with missing values were excluded from relevant analyses. After processing the original FAERS dataset, we created a dataset caused by the reports of B/F/TAF.

### 2.3 Data standardization

Adverse event terms in FAERS are coded using the Medical Dictionary for Regulatory Activities (MedDRA). For this analysis, we mapped all reported events to MedDRA version 27.0, utilizing the hierarchical structure of Preferred Terms (PTs) and System Organ Classes (SOCs) to classify and standardize event descriptions. (Sakaeda et al., 2013; Omar et al., 2021).

### 2.4 Data mining

To detect safety signals, we employed four complementary disproportionality methods: (1) reporting odds ratio (ROR), (2) proportional reporting ratio (PRR) with chi-square statistics, (3) Bayesian confidence propagation neural network (BCPNN) measuring the information component (IC), and (4) multi-item gamma Poisson shrinker (MGPS) calculating the empirical Bayesian geometric mean (EBGM). This combination of frequentist and Bayesian approaches enhances signal detection robustness. Signal detection criteria were defined as follows: ROR signal if the lower bound of the 95% confidence interval exceeded 1; PRR signal if PRR >2 and χ^2^ > 4; BCPNN signal if the lower limit of the information component (IC025) was >0; and MGPS signal if EBGM05 (the lower bound of the 95% CI for the empirical Bayesian geometric mean) exceeded 2 (van Puijenbroek et al., 2002; Ooba and Kubota, 2010; Szarfman et al., 2002; Evans et al., 2001). All statistical analyses and graphical representations were performed using Microsoft Excel (version 2021) and R (version 4.3.3).

## 3 Results

### 3.1 Basic information on safety signals

The study cohort consisted of 6816 B/F/TAF safety signal reports. Of these, reports from male patients accounted for 68.9% (4,701 cases). In terms of age distribution, patients aged 18–65 years submitted the majority of reports, comprising 49.1% (3,345 cases), whereas reports from children under 18 years were the fewest, accounting for only 1.2% (80 cases). Among the categories of reporters, consumers submitted the highest proportion of reports at 30.3% (2065 cases), followed by medical doctor at 25.1% (1717 cases). Geographically, the United States had the highest number of reports, making up 74.2% (5,062 cases). Detailed reporting information is presented in Table 1.

**TABLE 1:** Basic information on B/F/TAF-related adverse drug signals.

Category	Subcategory	Reported cases [n (%)]
Gender	Male	4,701 (68.9%)
	Female	1,587 (23.2%)
	Unknown	527 (7.7%)
Age (years)	<18	80 (1.2%)
	≥18-<65	3,345 (49.1%)
	≥65	455 (6.7%)
	Unknown	2,935 (43%)
Reporter	Consumer	2065 (30.3%)
	Medical doctor	1717 (25.1%)
	Healthcare professional	1,311 (19.2%)
	Other	1,600 (23.5%)
	Unknown	122 (1.7%)
Reporting country (top 3)	United States	5,062 (74.2%)
	France	272 (3.9%)
	Portugal	175 (2.5%)

### 3.2 System Organ Classes affected by safety signals

A total of 13,853 safety signal reports for B/F/TAF affected 27 SOCs. As shown in Figure 1, the highest number of signals were related to “Injury, poisoning and procedural complications”, with 1792 cases, accounting for the largest proportion of all safety signals. This was followed by “general disorders and administration site conditions”, with 1726 cases, and “investigations”, with 1,499 cases. According to the statistics, the significant SOCs that met four criteria were “congenital, familial and genetic disorders”, and “pregnancy, puerperium and perinatal conditions”. Also, “social circumstances”, “immune system disorders”, “hepatobiliary disorders”, “metabolism and nutrition disorders”, “renal and urinary disorders”, “surgical and medical procedures”, “psychiatric disorders”, “infections and infestations”, “investigations”, and “injury, poisoning and procedural complications” were significant SOCs that at least one of the four indices met the criteria. Detailed information is presented in Figure 2.

**FIGURE 1:**
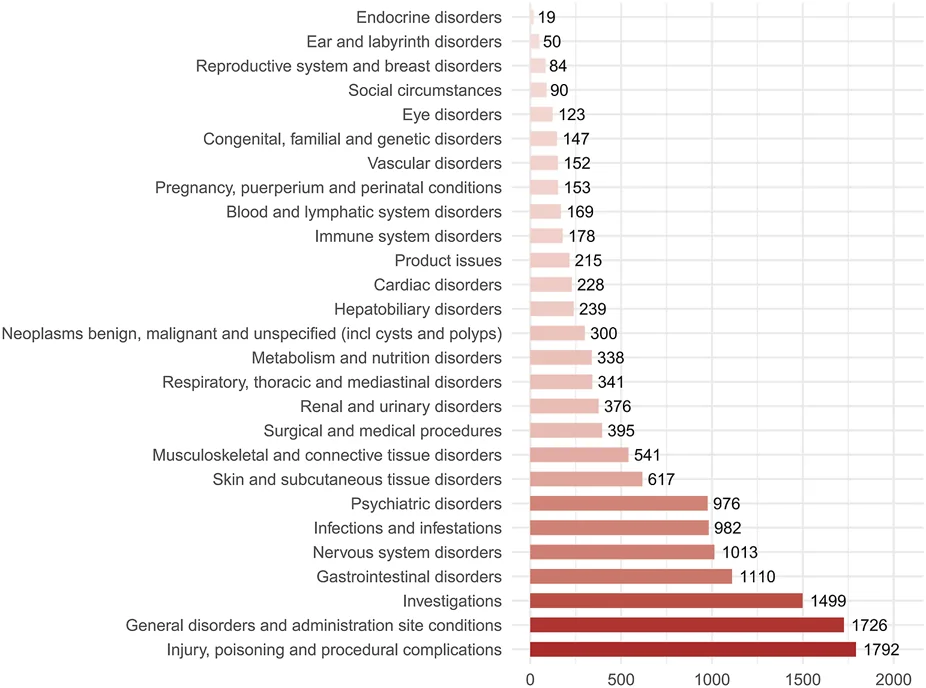
B/F/TAF adverse drug signals affecting SOCs.

**FIGURE 2:**
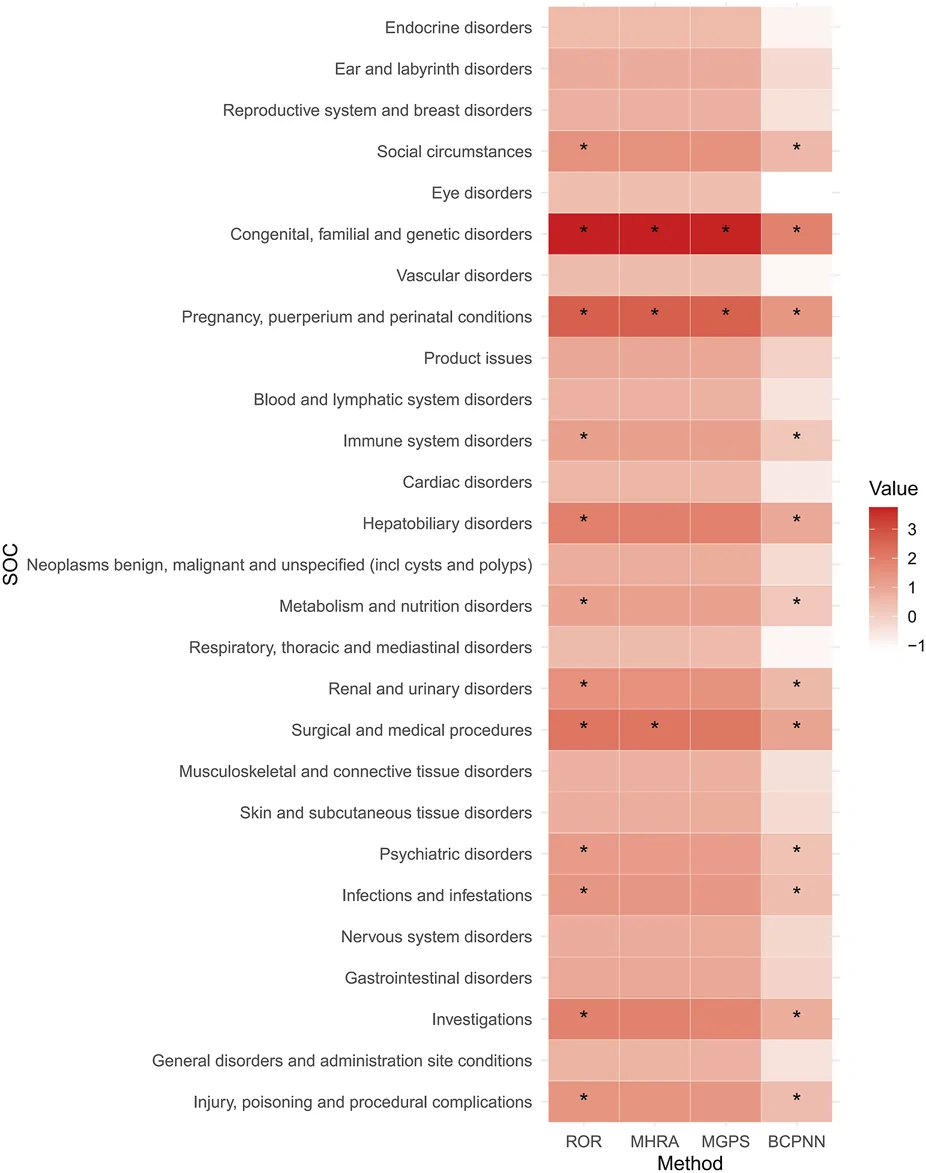
Signal strength of reports of B/F/TAF at the SOC level.

### 3.3 Adverse drug signal mining results

From the analysis of 13,853 B/F/TAF safety signal reports, 179 valid signals were identified. The top 20 preferred terms (PTs) were ranked in descending order based on the number of safety signal reports and reporting odds ratio (ROR) values. Table 2 presents the detailed information. Of these 20 PTs, several are medication process errors (e.g., product dose omission, product dispensing error, intentional dose omission, treatment noncompliance) or treatment monitoring parameters (e.g., viral load increased, CD4 lymphocytes decreased), which should not be classified as biological adverse drug reactions. The remaining PTs represent potential safety signals warranting further investigation. The top three PTs by the number of reports were weight increase (408 cases), product dose omission issue (242 cases), and maternal exposure during pregnancy (202 cases). The top three PTs by ROR values were viral load increased (ROR 160.14), CD4 lymphocytes decreased (ROR 124.60), and IRIS (ROR 88.12). Notably, 75% (15 out of 20) of the PTs have not been previously reported, though some represent medication process errors or treatment monitoring parameters rather than pharmacological adverse reactions.

**TABLE 2:** Top 20 PTs by number of adverse drug signals for B/F/TAF.

Preferred term (PT)	Cases	ROR (95%CI)	PRR (χ2)	IC (IC025)	EBGM (EBGM05)
Weight increased	408	8.53 (7.73-9.41)	8.31 (2626.19)	3.05 (2.88)	8.29 (7.51)
Product dose omission issue*	242	4.62 (4.07-5.24)	4.56 (673.34)	2.19 (1.98)	4.55 (4.01)
Maternal exposure during pregnancy*	202	11.17 (9.72-12.83)	11.02 (1837.22)	3.46 (3.18)	10.99 (9.56)
Viral load increased	190	160.14 (138.51-185.14)	154.13 (28838.94)	7.27 (7.08)	153.87 (134.42)
Product storage error*	174	9.95 (8.57-11.56)	9.84 (1380.38)	3.3 (3)	9.82 (8.45)
Product use issue*	106	2.64 (2.18-3.19)	2.63 (107.01)	1.39 (1.09)	2.63 (2.17)
Foetal exposure during pregnancy*	106	5.94 (4.91-7.20)	5.91 (431.99)	2.56 (2.22)	5.9 (4.87)
Immune reconstitution inflammatory syndrome	97	88.12 (72.02-107.83)	87.51 (8121.45)	6.42 (5.2)	85.69 (70.03)
Product dispensing error*	95	21.31 (17.41-26.09)	21.17 (1816.7)	4.4 (3.83)	21.06 (17.21)
Intentional dose omission*	85	17.17 (13.87-21.27)	17.08 (1281.49)	4.09 (3.53)	17.01 (13.74)
Neoplasm malignant*	81	5.52 (4.43-6.86)	5.49 (297.36)	2.46 (2.06)	5.48 (4.41)
Treatment noncompliance*	74	6.33 (5.03-7.95)	6.3 (329.65)	2.65 (2.22)	6.29 (5.01)
Dysphagia*	71	3.38 (2.68-4.27)	3.37 (118.44)	1.75 (1.36)	3.37 (2.67)
Diabetes mellitus*	63	3.62 (2.82-4.63)	3.61 (118.66)	1.85 (1.43)	3.6 (2.81)
Drug resistance*	62	11.00 (8.57-14.12)	10.95 (559.47)	3.45 (2.87)	10.93 (8.51)
CD4 lymphocytes decreased	61	124.60 (96.53-160.84)	124.06 (7225.68)	6.91 (4.99)	120.41 (93.28)
Product dose omission*	54	3.33 (2.55-4.35)	3.32 (87.75)	1.73 (0.28)	3.32 (2.54)
Circumstance or information capable of leading to medication error*	52	7.05 (5.37-9.26)	7.03 (268.53)	2.81 (0.26)	7.02 (5.34)
Abortion spontaneous*	51	5.61 (4.26-7.38)	5.59 (191.99)	2.48 (1.96)	5.58 (4.24)
Blood creatinine increased	50	3.37 (2.55-4.45)	3.36 (82.89)	1.75 (1.28)	3.36 (2.54)

PTs, marked with * include not only potential adverse reactions but also medication process errors (e.g., dose omission, dispensing errors) and treatment monitoring parameters (e.g., viral load increased, CD4 lymphocytes decreased).

### 3.4 Onset time of events

The onset times of B/F/TAF-associated adverse signals were collected from the database. Excluding unreported or unknown onset time reports, a total of 2064 safety signals (30.28%, data available in 2064/6,816) reported onset time and the median onset time was 60 days (interquartile range [IQR] 14–237 days). As shown in Figure 3, results indicated that the onset time of safety signals were variable, most of the cases occurred within the first (n = 783, 37.94%), second (n = 257, 12.45%) and third month (n = 150, 7.27%) after B/F/TAF initiation. Notably, safety signals might still occur after 1 year of B/F/TAF treatment with 17.49% as illustrated in our data.

**FIGURE 3:**
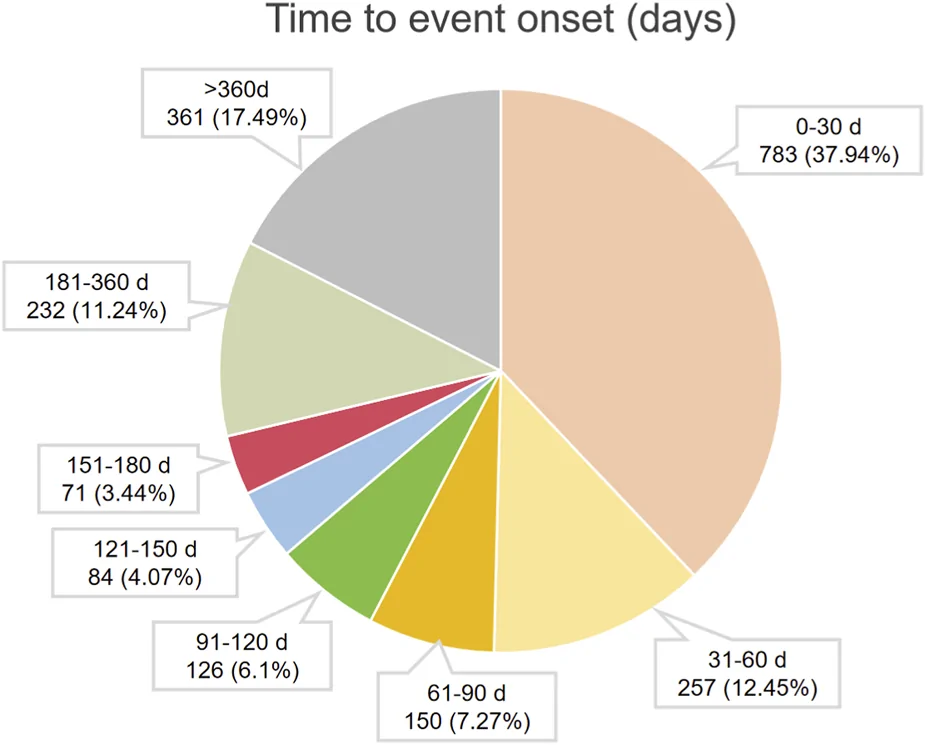
Time-to-onset of adverse signals.

## 4 Discussion

This study systematically evaluates the safety profile of B/F/TAF based on data from the global spontaneous reporting system. Utilizing a substantial number of safety signal reports from the FAERS, we thoroughly explored the potential adverse drug events induced by B/F/TAF through disproportionality analysis and further clinical priority assessment.

Our findings indicate that males reported significantly more safety signal reports compared to females (68.9% vs. 23.2%). According to statistics from the Joint United Nations Programme on HIV/AIDS (UNAIDS, 2025), over 73% of new HIV infections in 2023 in regions outside of sub-Saharan Africa were among males. This significant gender disparity reflects the higher prevalence of HIV among males globally and may also suggest differences in healthcare-seeking behavior or treatment access between sexes (Pugatch et al., 2000). Regarding age, nearly half of all reports (49.1%) originated from patients aged 18–65 years, a finding consistent with the typical demographic of PLWH receiving antiretroviral therapy. Strikingly, reports involving children under 18 years and elderly patients (≥65 years) were remarkably sparse, accounting for only 1.2% and 6.7% of the total, respectively. While this may partially mirror lower prescription rates in pediatric and geriatric populations, it also exposes a critical evidence gap regarding the real-world safety profile of B/F/TAF in these vulnerable subgroups, where pharmacokinetic variability, polypharmacy, and age-related comorbidities may substantially modify drug tolerability (Rodriguez et al., 2024; Toledo et al., 2023). In addition, the proportion of missing age data is extremely high (43%), and there is an urgent need to obtain more granular demographic information through targeted pharmacovigilance initiatives.

Among the SOCs that met all four disproportionality criteria, “Pregnancy, puerperium and perinatal conditions” and “Congenital, familial and genetic disorders” emerged as particularly noteworthy. At the PT level, “maternal exposure during pregnancy”, “foetal exposure during pregnancy”, and “spontaneous abortion” all demonstrated robust signal generation. The ROR for “maternal exposure during pregnancy” was substantially higher than that for many established adverse drug signals, reflecting pharmacovigilance reporting of drug exposure during pregnancy rather than clinical adverse events. Notably, “spontaneous abortion” also generated a signal (n = 51, ROR 5.61), which warrants attention but must be interpreted in context. Preliminary pharmacokinetic data support once-daily B/F/TAF without dose adjustment during pregnancy (Holt et al., 2024), and available pregnancy outcome data have not shown congenital malformations above the background rate (Olivero et al., 2025). The FAERS signals identified in our study, while hypothesis-generating rather than causal, call for continued vigilance and robust pregnancy registries to better characterize the reproductive safety profile of B/F/TAF.

Several other SOCs meeting at least one disproportionality criterion deserve careful interpretation due to their clinical relevance. The “Metabolism and nutrition disorders” SOC, driven largely by “weight increased” and “diabetes mellitus”, corroborates growing concerns regarding INSTI-associated metabolic complications. A recent META-INSTI analysis of FAERS data reported RORs for hyperglycaemia/new-onset diabetes mellitus and weight gain for bictegravir of 1.23 (1.10–1.37) and 6.82 (5.50–8.41), respectively, consistent with our findings (Murray et al., 2023). However, long-term phase 3 trial data and real-world BICSTaR cohorts suggest weight gain is most pronounced in the first year and in individuals with more advanced HIV at baseline, supporting the ‘return to health’ hypothesis rather than a purely drug-driven effect (Daar et al., 2025; Hocqueloux et al., 2025; Inciarte et al., 2025). The “Renal and urinary disorder” SOC, represented by “blood creatinine increased” (n = 50, ROR 3.37), reflects the known renal tubular effect of TAF without necessarily indicating glomerular injury, consistent with the established renal safety profile of TAF (Gupta et al., 2019; Liang et al., 2025).

Finally, several strong signals identified in our analysis, particularly “viral load increased”, “CD4 lymphocytes decreased” and “drug resistance”, likely reflect treatment failure, poor adherence, or advanced HIV disease rather than direct B/F/TAF toxicity. The high ROR values for these PTs likely reflect their routine monitoring in HIV care and reporting when treatment targets are not met, rather than a causal relationship with B/F/TAF. Similarly, IRIS represents immune reconstitution pathology following viral suppression rather than a drug-specific adverse effect: as B/F/TAF rapidly suppresses viral replication and restores CD4 counts, the reconstituted immune system may mount inflammatory responses against opportunistic pathogens (Tappuni, 2011). This signal serves as a reminder that IRIS remains a clinically important complication following ART initiation, particularly in patients with advanced immunodeficiency at baseline (Müller et al., 2010). It is critical to distinguish between pharmacovigilance signals identified through disproportionality analysis and confirmed adverse drug reactions.

Our analysis of time-to-onset data revealed that, among 2,064 B/F/TAF-associated adverse signals with documented onset times (30.28% of all reports), the median time to onset was 60 days, with the majority of events occurring within the first 3 months of treatment initiation. Notably, the first month alone accounted for 37.94% of all reported events, followed by 12.45% (n = 257) in the second month and 7.27% (n = 150) in the third month. This early clustering of adverse signals is consistent with findings from a contemporaneous FAERS-based pharmacovigilance study of B/F/TAF, which reported that most adverse signals with valid time-to-onset data occurred within the first month (Xie et al., 2026). The high proportion of early-onset events underscores the first 90 days of therapy as a critical window for intensified safety monitoring. Importantly, 17.49% of events were reported after 1 year of therapy, challenging the assumption that safety surveillance can be safely relaxed after the initial phase. Long-term vigilance should focus on metabolic and cardiovascular risks (weight gain, diabetes, dyslipidemia) requiring periodic glucose, lipid, and risk-score monitoring (Daar et al., 2025). Hepatobiliary events, including HBV flares and steatosis, may occur at varying times after initiation or withdrawal (Lindhardt et al., 2026). Moreover, treatment noncompliance and intentional dose omission, new ADRs in our top 20, may emerge due to pill fatigue or perceived wellness, reinforcing the need for sustained adherence counseling (Maggiolo et al., 2024).

## 5 Limitations

Several limitations warrant consideration. First, confounding by indication is inherent to FAERS analyses, as disease severity and prior ART exposure may influence treatment selection and reported outcomes. Second, concomitant medications and prior regimen switches may contribute to events through drug–drug interactions, withdrawal effects, or metabolic changes unrelated to B/F/TAF. Third, the higher baseline prevalence of comorbidities (e.g., metabolic syndrome, renal impairment, psychiatric disorders) in PLWH may predispose to certain reports. Fourth, reporting bias may preferentially capture more clinically significant or novel events. These factors preclude causal attribution and underscore the hypothesis-generating nature of our findings. Additionally, the absence of a comparator group precludes determination of whether observed signals are specific to B/F/TAF or reflect class effects of INSTIs, as weight gain and metabolic changes have been reported across multiple INSTIs. Finally, the time-to-onset analysis is limited by valid onset dates available in only 30.28% of reports, potentially introducing selection bias.

## 6 Conclusion

This pharmacovigilance analysis of real-world FAERS data identified a spectrum of statistical associations for B/F/TAF, including potential biological adverse reactions, medication process errors, and treatment monitoring parameters. Several of these signals are not currently reflected in the product label, though some represent medication-use issues or disease-related events rather than drug-attributable adverse reactions. These observations should serve primarily to inform and prioritize future prospective investigations, rather than to guide clinical decision-making directly. Ultimately, continuous pharmacovigilance remains indispensable to fully characterize the long-term safety profile of B/F/TAF in the diverse and aging population of people living with HIV.

## Data Availability

The original contributions presented in the study are included in the article/supplementary material, further inquiries can be directed to the corresponding author.
